# Prevalence of Disabilities and Health Care Access by Disability Status and Type Among Adults — United States, 2016

**DOI:** 10.15585/mmwr.mm6732a3

**Published:** 2018-08-17

**Authors:** Catherine A. Okoro, NaTasha D. Hollis, Alissa C. Cyrus, Shannon Griffin-Blake

**Affiliations:** 1 Division of Human Development and Disability, National Center on Birth Defects and Developmental Disabilities, CDC.

## Abstract

Persons with disabilities face greater barriers to health care than do those without disabilities (*1*). To identify characteristics of noninstitutionalized adults with six specific disability types (hearing, vision, cognition, mobility, self-care, and independent living),* and to assess disability-specific disparities in health care access, CDC analyzed 2016 Behavioral Risk Factor Surveillance System (BRFSS) data. The prevalences of disability overall and by disability type, and access to health care by disability type, were estimated. Analyses were stratified by three age groups: 18–44 years (young adults), 45–64 years (middle-aged adults), and ≥65 years (older adults). Among young adults, cognitive disability (10.6%) was the most prevalent type. Mobility disability was most prevalent among middle-aged (18.1%) and older adults (26.9%). Generally, disability prevalences were higher among women, American Indians/Alaska Natives (AI/AN), adults with income below the federal poverty level (FPL), and persons in the South U.S. Census region. Disability-specific disparities in health care access were prevalent, particularly among young and middle-aged adults. These data might inform public health programs of the sociodemographic characteristics and disparities in health care access associated with age and specific disability types and guide efforts to improve access to care for persons with disabilities.

BRFSS is an ongoing state-based, random-digit–dialed telephone survey of noninstitutionalized U.S. adults aged ≥18 years.^†^ The median survey response rate among the 50 states and the District of Columbia in 2016 was 47.0%.^§^ The 2016 BRFSS survey included questions about six disability types (hearing, vision, cognition, mobility, self-care, and independent living).^¶^ Respondents were identified as having one of the disability types if they answered “yes” to the relevant question. Persons who responded “yes” to at least one disability question were identified as having any disability. Persons who responded “no” to all six questions were identified as having no disability. Missing responses and respondents who answered “don’t know” or who declined to answer were excluded. Four health care access measures (i.e., health insurance coverage, having a usual health care provider, receipt of a routine check-up within the past year, and having an unmet health care need because of cost) were included.** Prevalences (with 95% confidence intervals) were calculated for any disability and disability type by sex, race/ethnicity,^††^ FPL,^§§^ and U.S. Census region, and for health care access measures, by disability status and types. All analyses were stratified by age group (18–44, 45–64, and ≥65 years). Analyses accounted for the complex sampling design.

One in four noninstitutionalized U.S. adults (25.7%, representing an estimated 61.4 million persons) reported any disability (Table 1) (Figure). Mobility was the most prevalent disability type (13.7%), followed by cognition (10.8%), independent living (6.8%), hearing (5.9%), vision (4.6%), and self-care (3.7%). Prevalences of any disability, hearing, mobility, and independent living disabilities were higher among older adults, whereas prevalence of cognitive disability was highest among middle-aged (11.9%) and young adults (10.6%), and lowest among older adults (9.5%). Among middle-aged and older adults, the prevalences of vision disability (6.1% and 6.6%, respectively) and self-care disability (5.5% in both) were similar. Among all age groups, the prevalences of any disability and of each type were higher among women than among men, with the exceptions of hearing and self-care. The reported prevalence of hearing disability was higher among men than among women for all age groups (young adults: men = 2.4% versus women = 1.6%; middle-aged adults: 7.6% versus 4.2%; and older adults: 19.4% versus 11.3%), and the reported prevalences of self-care disability were approximately the same. Generally, among young and middle-aged adults, the highest prevalences of any disability and of each type were reported among AI/AN and persons in the “other race/multiracial” group, whereas the lowest prevalences were reported among Asians. Among older adults, approximately half of AI/AN (54.9%), Hispanics (50.5%), and persons in the “other race/multiracial” group (49.9%) reported any disability. Within each age group, the prevalences of any and each disability type declined with decreasing poverty. Across all age groups, higher prevalences of any disability and of each type were generally reported in the South compared with other U.S. Census regions.

**TABLE 1:** Weighted unadjusted prevalence estimates of disability among adults, by type of disability* and selected characteristics — Behavioral Risk Factor Surveillance System, 2016

Characteristic	No. of respondents^†,§^	Type of disability^¶^						
		Hearing	Vision	Cognition	Mobility	Self-care	Independent living	Any
		% (95% CI)	% (95% CI)	% (95% CI)	% (95% CI)	% (95% CI)	% (95% CI)	% (95% CI)
**Total (18–44 yrs)**	**121,674**	**2.0 (1.8–2.1)**	**2.7 (2.5–2.9)**	**10.6 (10.3–10.9)**	**4.8 (4.6–5.0)**	**1.7 (1.5–1.8)**	**4.5 (4.3–4.7)**	**16.6 (16.2–16.9)**
**Sex**								
Men	58,295	2.4 (2.2–2.6)	2.4 (2.2–2.6)	9.5 (9.0–9.9)	4.0 (3.8–4.3)	1.6 (1.4–1.8)	3.5 (3.3–3.8)	15.2 (14.7–15.7)
Women	63,356	1.6 (1.4–1.7)	3.0 (2.8–3.3)	11.7 (11.3–12.2)	5.6 (5.3–5.9)	1.7 (1.6–1.9)	5.5 (5.2–5.8)	17.9 (17.4–18.5)
Race/Ethnicity**								
White	80,322	2.0 (1.9–2.2)	2.2 (2.0–2.4)	10.9 (10.5–11.2)	4.5 (4.3–4.8)	1.6 (1.4–1.7)	4.8 (4.5–5.0)	16.3 (16.2–16.9)
Black	11,837	1.4 (1.2–1.7)	3.6 (3.1–4.2)	11.1 (10.2–12.0)	6.6 (6.0–7.4)	2.1 (1.7–2.6)	4.7 (4.1–5.5)	18.1 (17.0–19.3)
Hispanic	16,297	2.1 (1.8–2.5)	3.7 (3.3–4.3)	10.3 (9.5–11.1)	5.0 (4.5–5.5)	1.6 (1.4–1.9)	4.0 (3.5–4.5)	17.6 (16.6–18.5)
AI/AN	2,255	3.5 (2.4–5.0)	3.8 (2.8–5.2)	18.8 (15.9–22.1)	8.6 (6.8–10.9)	2.3 (1.5–3.7)^††^	8.4 (6.6–10.8)	27.7 (24.4–31.2)
Asian	4,754	0.8 (0.5–1.3)^††^	1.3 (0.9–1.8)	4.5 (3.7–5.6)	1.1 (0.7–1.6)	N/A^§§^	1.1 (0.8–1.6)	7.2 (6.2–8.4)
Other race/Multiracial	4,508	3.7 (2.8–4.9)	3.4 (2.7–4.3)	16.0 (14.1–18.1)	7.5 (6.3–9.1)	3.0 (2.2–4.1)	8.4 (6.9–10.1)	24.9 (22.7–27.3)
**Federal poverty level (FPL)** ^¶¶^								
<100% of FPL (poor)	18,824	3.3 (2.9–3.7)	5.3 (4.8–5.8)	18.2 (17.3–19.1)	10.4 (9.7–11.1)	3.5 (3.1–3.9)	9.4 (8.7–10.1)	27.8 (26.7–28.9)
≥100%–<200% of FPL (near poor)	24,116	2.1 (1.8–2.3)	3.2 (2.8–3.6)	12.8 (12.1–13.6)	5.7 (5.2–6.2)	2.0 (1.7–2.3)	5.8 (5.3–6.3)	20.1 (19.2–21.0)
≥200% of FPL (not poor)	59,273	1.3 (1.2–1.4)	1.3 (1.1–1.5)	5.5 (5.2–5.9)	2.0 (1.9–2.3)	0.7 (0.6–0.8)	1.7 (1.5–1.9)	9.3 (8.9–9.7)
Unknown	19,461	2.3 (2.0–2.7)	3.2 (2.8–3.7)	13.4 (12.6–14.2)	5.4 (4.9–5.9)	2.0 (1.7–2.3)	5.5 (5.0–6.0)	19.9 (19.0–20.9)
**U.S. Census region**								
Northeast	23,348	1.4 (1.2–1.7)	2.4 (2.1–2.7)	9.5 (8.9–10.2)	4.2 (3.8–4.6)	1.5 (1.3–1.8)	4.4 (3.9–4.8)	15.3 (14.5–16.1)
Midwest	29,963	2.0 (1.7–2.2)	2.1 (1.9–2.4)	10.9 (10.3–11.5)	4.9 (4.5–5.3)	1.8 (1.5–2.0)	4.6 (4.2–5.0)	16.4 (15.7–17.1)
South	39,745	2.2 (2.0–2.5)	3.4 (3.1–3.8)	11.5 (11.0–12.1)	5.6 (5.3–6.0)	1.8 (1.6–2.0)	4.8 (4.5–5.2)	18.1 (17.5–18.8)
West	28,618	1.9 (1.7–2.2)	2.3 (2.1–2.7)	9.5 (8.9–10.1)	3.9 (3.5–4.3)	1.4 (1.2–1.7)	4.1 (3.7–4.5)	15.2 (14.5–15.9)
**Total (45–64 yrs)**	**174,413**	**5.9 (5.6–6.1)**	**6.1 (5.9–6.4)**	**11.9 (11.6–12.2)**	**18.1 (17.7–18.5)**	**5.5 (5.3–5.7)**	**8.2 (7.9–8.5)**	**28.6 (28.2–29.1)**
**Sex**								
Men	76,489	7.6 (7.3–8.0)	5.8 (5.4–6.2)	10.2 (9.8–10.6)	16.1 (15.5–16.6)	5.5 (5.2–5.9)	6.9 (6.6–7.4)	27.1 (26.5–27.7)
Women	97,910	4.2 (3.9–4.4)	6.4 (6.1–6.8)	13.5 (13.0–13.9)	20.1 (19.5–20.6)	5.4 (5.2–5.8)	9.4 (9.0–9.8)	30.1 (29.5–30.7)
Race/Ethnicity**								
White	135,958	5.9 (5.7–6.2)	4.6 (4.4–4.8)	10.8 (10.5–11.1)	16.2 (15.9–16.6)	4.7 (4.5–4.9)	7.4 (7.1–7.6)	26.2 (25.8–26.7)
Black	14,851	5.0 (4.4–5.8)	9.6 (8.7–10.6)	14.5 (13.5–15.6)	25.3 (24.0–26.6)	7.9 (7.1–8.7)	10.5 (9.7–11.4)	35.5 (34.1–37.0)
Hispanic	10,400	6.0 (5.1–7.0)	11.2 (10.0–12.5)	14.4 (13.2–15.7)	21.8 (20.4–23.4)	7.4 (6.5–8.4)	9.5 (8.6–10.6)	35.5 (33.7–37.2)
AI/AN	2,910	14.3 (11.7–17.2)	11.5 (9.7–13.6)	23.9 (20.7–27.3)	33.3 (29.9–36.9)	10.3 (8.4–12.7)	16.6 (14.0–19.5)	49.2 (45.5–52.8)
Asian	2,836	2.9 (1.9–4.4)^††^	N/A^§§^	6.4 (4.6–8.8)	7.6 (5.7–10.2)	N/A^§§^	4.4 (2.7–7.1)^††^	15.3 (12.5–18.4)
Other race/Multiracial	4,216	8.8 (7.2–10.8)	9.3 (7.5–11.6)	20.4 (16.6–24.9)	28.6 (24.7–32.9)	11.3 (7.9–16.0)	17.1 (13.4–21.7)	41.6 (37.6–45.6)
**Federal poverty level (FPL)** ^¶¶^								
<100% of FPL (poor)	16,128	9.0 (8.2–9.8)	16.4 (15.2–17.6)	30.0 (28.5–31.5)	42.3 (40.7–44.0)	15.7 (14.5–17.0)	22.8 (21.4–24.2)	57.9 (56.3–59.6)
≥100%–<200% of FPL (near poor)	30,911	8.7 (8.0–9.4)	9.9 (9.1–10.8)	18.5 (17.6–19.3)	29.1 (28.1–30.1)	9.1 (8.5–9.7)	13.3 (12.6–14.0)	44.5 (43.3–45.7)
≥200% of FPL (not poor)	102,245	4.1 (3.8–4.3)	2.4 (2.2–2.6)	5.4 (5.1–5.7)	8.9 (8.5–9.3)	2.1 (1.9–2.3)	3.2 (3.0–3.5)	16.6 (16.1–17.1)
Unknown	25,129	6.8 (6.3–7.4)	7.5 (6.8–8.2)	14.3 (13.4–15.1)	20.9 (19.9–21.8)	5.9 (5.4–6.5)	9.6 (9.0–10.3)	31.9 (30.8–33.1)
**U.S. Census region**								
Northeast	37,594	4.8 (4.4–5.3)	4.9 (4.5–5.4)	10.2 (9.6–10.8)	16.0 (15.2–16.8)	4.6 (4.2–5.1)	7.3 (6.8–7.8)	25.6 (24.7–26.5)
Midwest	42,247	5.9 (5.6–6.3)	5.1 (4.7–5.5)	10.9 (10.4–11.5)	16.9 (16.3–17.6)	5.0 (4.6–5.4)	7.3 (6.9–7.7)	27.0 (26.3–27.8)
South	57,726	6.7 (6.3–7.2)	7.6 (7.1–8.1)	13.7 (13.1–14.3)	21.5 (20.9–22.2)	6.6 (6.2–7.1)	9.6 (9.1–10.1)	32.7 (31.9–33.5)
West	36,846	5.2 (4.7–5.6)	5.6 (5.1–6.2)	11.1 (10.4–11.8)	15.3 (14.5–16.1)	4.8 (4.3–5.3)	7.4 (6.8–8.1)	25.8 (24.9–26.8)
**Total (≥65 yrs)**	**162,724**	**14.9 (14.5–15.3)**	**6.6 (6.4–6.9)**	**9.5 (9.2–9.9)**	**26.9 (26.5–27.4)**	**5.5 (5.2–5.8)**	**9.8 (9.4–10.1)**	**41.7 (41.1–42.2)**
**Sex**								
Men	64,224	19.4 (18.7–20.1)	6.2 (5.8–6.7)	8.8 (8.3–9.4)	22.8 (22.1–23.5)	5.1 (4.7–5.5)	6.5 (6.1–7.0)	40.9 (40.0–41.7)
Women	98,488	11.3 (10.8–11.7)	7.0 (6.6–7.3)	10.1 (9.7–10.6)	30.3 (29.6–30.9)	5.8 (5.4–6.2)	12.3 (11.8–12.8)	42.3 (41.6–43.0)
Race/Ethnicity**								
White	138,816	15.5 (15.1–15.9)	5.9 (5.6–6.2)	8.4 (8.1–8.7)	25.5 (25.0–25.9)	4.6 (4.3–4.8)	8.8 (8.5–9.1)	40.2 (39.6–40.7)
Black	10,022	10.2 (8.7–11.9)	8.8 (7.8–10.0)	12.3 (11.0–13.7)	33.6 (31.6–35.6)	8.4 (7.3–9.7)	13.3 (11.9–14.8)	46.7 (44.6–48.8)
Hispanic	4,583	14.0 (12.1–16.3)	10.8 (9.2–12.5)	15.5 (13.4–17.7)	33.3 (30.7–36.1)	9.4 (7.8–11.3)	15.4 (13.4–17.6)	50.5 (47.7–53.4)
AI/AN	1,702	25.3 (21.2–29.9)	8.9 (6.9–11.5)	17.0 (13.8–20.7)	37.5 (33.0–42.2)	10.0 (7.6–13.1)	14.9 (12.1–18.2)	54.9 (50.0–59.8)
Asian	1,739	9.6 (5.7–15.7)^††^	N/A^§§^	9.4 (5.6–15.4)^††^	22.5 (16.7–29.6)	N/A^§§^	5.1 (3.0–8.6)^††^	34.8 (28.2–42.1)
Other race/Multiracial	3,073	17.9 (14.7–21.6)	8.5 (6.6–11.0)	14.4 (11.9–17.3)	34.6 (30.9–38.6)	8.8 (6.6–11.5)	12.9 (10.1–16.4)	49.9 (45.8–54.0)
**Federal poverty level (FPL)** ^¶¶^								
<100% of FPL (poor)	7,962	18.1 (16.2–20.1)	13.7 (12.1–15.6)	18.2 (16.5–20.0)	43.5 (41.1–45.9)	12.0 (10.6–13.7)	19.5 (17.8–21.4)	59.6 (57.1–62.0)
≥100%–<200% of FPL (near poor)	41,124	17.4 (16.6–18.3)	9.3 (8.7–10.0)	13.2 (12.4–14.0)	36.4 (35.3–37.5)	7.8 (7.1–8.5)	13.7 (12.9–14.5)	53.1 (52.0–54.1)
≥200% of FPL (not poor)	79,774	12.8 (12.2–13.3)	4.1 (3.7–4.4)	5.5 (5.1–5.9)	18.7 (18.0–19.3)	3.0 (2.7–3.3)	5.1 (4.8–5.5)	31.9 (31.1–32.6)
Unknown	33,864	15.5 (14.7–16.4)	6.8 (6.3–7.3)	11.2 (10.5–12.0)	28.4 (27.4–29.5)	6.0 (5.4–6.8)	12.0 (11.2–12.8)	43.7 (42.5–44.8)
**U.S. Census region**								
Northeast	31,466	12.9 (12.1–13.8)	5.7 (5.1–6.3)	8.2 (7.5–9.0)	26.2 (25.1–27.3)	5.2 (4.6–5.9)	9.2 (8.5–10.0)	39.3 (38.1–40.5)
Midwest	39,575	15.0 (14.4–15.6)	5.9 (5.5–6.4)	8.2 (7.8–8.7)	25.2 (24.5–26.0)	4.7 (4.3–5.1)	9.0 (8.5–9.5)	40.3 (39.4–41.1)
South	56,913	15.8 (15.1–16.5)	7.8 (7.3–8.3)	11.1 (10.6–11.7)	28.8 (28.0–29.6)	6.0 (5.5–6.4)	11.0 (10.4–11.5)	44.3 (43.4–45.2)
West	34,770	14.9 (13.9–15.9)	6.2 (5.5–7.0)	9.1 (8.3–10.1)	26.1 (24.8–27.5)	5.6 (4.9–6.5)	8.8 (8.1–9.7)	40.4 (39.0–41.8)
**Total (all age groups)**	**458,811**	**5.9 (5.7–6.0)**	**4.6 (4.5–4.8)**	**10.8 (10.6–11.0)**	**13.7 (13.5–13.9)**	**3.7 (3.6–3.8)**	**6.8 (6.7–6.9)**	**25.7 (25.4–25.9)**

**Abbreviations:** AI/AN = American Indian/Alaska Native; CI = confidence interval; N/A = not available.

* Respondents were asked “Are you deaf or do you have serious difficulty hearing?” (hearing); “Are you blind or do you have serious difficulty seeing, even when wearing glasses?” (vision); “Because of a physical, mental, or emotional condition, do you have serious difficulty concentrating, remembering, or making decisions?” (cognition); “Do you have serious difficulty walking or climbing stairs?” (mobility); “Do you have difficulty dressing or bathing?” (self-care); and “Because of a physical, mental, or emotional condition, do you have difficulty doing errands alone such as visiting a doctor’s office or shopping?” (independent living). Respondents who declined to answer, reported “don’t know,” and other missing responses were excluded from the analyses.

^†^ Respondents with missing information on disability are not included; all groups might not add to the same respondent total or to the overall total.

^§^ Unweighted sample size.

^¶^ Each disability type might not be independent; a respondent might have two or more disability types.

** Persons in all racial groups were non-Hispanic. Persons who self-identified as Hispanic might have been of any race.

^†† ^Relative standard error = 0.20–0.30.

^§§^ Estimate not available because relative standard error >0.30.

^¶¶^ Poverty categories are based on the ratio of the respondent’s annual household income to the appropriate simplified 2015 federal poverty threshold (given family size: number of adults (1–14) in the household and number of children (≥0) in the household) defined by the U.S. Census Bureau. This ratio is multiplied by 100 to be expressed as a percentage, and federal poverty thresholds were then used to categorize respondents into four FPL categories: 1) <100% of FPL (poor), 2) ≥100%–<200% of FPL (near poor), 3) ≥200% of FPL (not poor), and 4) unknown.

**FIGURE:**
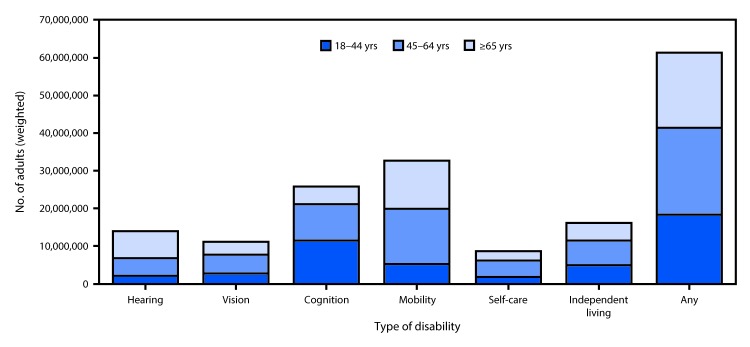
Estimated number of adults with any disability, by specific type of disability and age group — Behavioral Risk Factor Surveillance System, 2016

In 2016, for each disability type, prevalences of health insurance coverage, having a usual health care provider, and receiving a check-up during the preceding 12 months increased with increasing age group, whereas, with the exception of persons with a vision disability, the prevalence of having an unmet health care need because of cost decreased (Table 2). Young and middle-aged adults with a vision disability had the lowest prevalences of having health insurance coverage (74.9% and 81.3%, respectively), a usual health care provider (64.0% and 82.3%, respectively), and, among younger adults, of having received a check-up during the preceding 12 months (58.0%). Within these age groups, adults with a self-care disability had the highest prevalences of having health insurance coverage (83.1% and 88.8%, respectively) and a usual health care provider (76.3% and 89.0%, respectively), similar to middle-aged adults with an independent living disability (89.0%). The prevalences of having received a routine check-up during the past 12 months were higher among young adults with a mobility disability (69.1%) and middle-aged adults with a self-care disability (81.6%). Having a health care need that was unmet because of cost considerations was most prevalent among younger adults with an independent living disability (36.7%) and middle-aged adults with a vision disability (35.5%), and was least prevalent among younger and middle-aged adults with a hearing disability (31.2% and 24.1%, respectively). Most health care access measures were similar by disability type among older adults, with the exception of having an unmet health care need because of cost, which ranged from 7.3% (hearing) to 14.0% (self-care).

**TABLE 2:** Weighted unadjusted prevalence estimates for four health care access measures among adults with any disability, by age group and disability type* — Behavioral Risk Factor Surveillance System, 2016

Age group (yrs)	Characteristic	No. of respondents^†^	Type of disability^§^						
			Hearing	Vision	Cognition	Mobility	Self-care	Independent living	Any
			% (95% CI)	% (95% CI)	% (95% CI)	% (95% CI)	% (95% CI)	% (95% CI)	% (95% CI)
**18–44**	**Health insurance coverage**								
	Yes	16,446	76.9 (73.7–79.7)	74.9 (72.1–77.5)	78.6 (77.2–79.8)	82.0 (80.3–83.6)	83.1 (79.9–85.9)	81.2 (79.3–83.0)	78.9 (77.8–79.9)
	No	3,690	23.1 (20.1–26.3)	25.1 (22.5–27.9)	21.5 (20.2–22.8)	18.0 (16.4–19.7)	16.9 (14.1–20.2)	18.8 (17.0–20.7)	21.2 (20.1–22.2)
	**Usual health care provider**								
	Yes	14,188	64.4 (61.1–67.5)	64.0 (61.0–66.9)	66.1 (64.6–67.5)	74.1 (72.1–76.0)	76.3 (72.8–79.5)	70.4 (68.2–72.4)	66.3 (65.2–67.5)
	No	5,967	35.6 (32.5–38.9)	36.0 (33.1–39.0)	34.0 (32.5–35.4)	25.9 (24.0–27.9)	23.7 (20.5–27.2)	29.7 (27.6–31.8)	33.7 (32.6–34.9)
	**Unmet health care need because of cost during past 12 mos.**								
	Yes	6,234	31.2 (28.3–34.2)	34.8 (32.0–37.7)	33.4 (32.0–34.8)	35.6 (33.6–37.7)	36.2 (32.9–39.6)	36.7 (34.6–38.9)	31.4 (30.3–32.5)
	No	13,957	68.8 (65.8–71.7)	65.2 (62.3–68.0)	66.6 (65.2–68.0)	64.4 (62.3–66.4)	63.8 (60.4–67.1)	63.3 (61.1–65.4)	68.6 (67.5–69.7)
	**Routine check-up within past 12 mos.**								
	Yes	12,509	60.5 (57.3–63.7)	58.0 (54.9–61.0)	61.4 (59.9–62.9)	69.1 (67.0–71.1)	67.9 (64.2–71.4)	64.4 (62.1–66.5)	61.7 (60.5–62.9)
	No	7,324	39.5 (36.3–42.7)	42.0 (39.0–45.1)	38.6 (37.1–40.1)	30.9 (28.9–33.0)	32.1 (28.6–35.8)	35.7 (33.5–37.9)	38.3 (37.1–39.5)
**45–64**	**Health insurance coverage**								
	Yes	44,085	87.1 (85.4–88.6)	81.3 (79.3–83.1)	86.3 (85.2–87.4)	88.4 (87.6–89.2)	88.8 (87.4–90.2)	88.4 (87.2–89.5)	87.0 (86.3–87.7)
	No	4,918	13.0 (11.4–14.6)	18.7 (16.9–20.7)	13.7 (12.6–14.8)	11.6 (10.8–12.4)	11.2 (9.8–12.6)	11.6 (10.5–12.9)	13.0 (12.3–13.7)
	**Usual health care provider**								
	Yes	43,142	84.9 (83.1–86.4)	82.3 (80.4–84.1)	85.3 (84.1–86.4)	88.3 (87.5–89.1)	89.0 (87.6–90.2)	89.0 (87.9–90.0)	85.8 (85.1–86.5)
	No	5,835	15.2 (13.6–16.9)	17.7 (15.9–19.6)	14.7 (13.6–15.9)	11.7 (10.9–12.5)	11.0 (9.8–12.4)	11.0 (10.0–12.1)	14.2 (13.5–14.9)
	**Unmet health care need because of cost during past 12 mos.**								
	Yes	11,506	24.1 (22.4–25.9)	35.5 (33.3–37.8)	31.8 (30.5–33.2)	27.2 (26.2–28.3)	31.9 (29.9–34.1)	31.9 (30.2–33.6)	25.9 (25.1–26.8)
	No	37,472	75.9 (74.1–77.6)	64.5 (62.2–66.7)	68.2 (66.8–69.5)	72.8 (71.7–73.8)	68.1 (65.9–70.1)	68.1 (66.4–69.8)	74.1 (73.2–74.9)
	**Routine check-up within past 12 mos.**								
	Yes	37,876	74.5 (72.7–76.2)	75.0 (73.1–76.9)	76.8 (75.6–78.0)	80.3 (79.4–81.2)	81.6 (80.0–83.1)	80.9 (79.6–82.2)	77.0 (76.1–77.7)
	No	10,596	25.5 (23.8–27.3)	25.0 (23.1–26.9)	23.2 (22.0–24.4)	19.7 (18.8–20.6)	18.4 (16.9–20.0)	19.1 (17.8–20.4)	23.1 (22.3–23.9)
**≥65**	**Health insurance coverage**								
	Yes	65,481	97.9 (97.4–98.3)	97.0 (96.1–97.8)	97.4 (96.8–97.9)	97.7 (97.4–98.0)	97.7 (96.9–98.2)	97.0 (96.2–97.6)	97.8 (97.6–98.1)
	No	1,191	2.1 (1.7–2.6)	3.0 (2.2–3.9)	2.6 (2.1–3.2)	2.3 (2.0–2.6)	2.4 (1.8–3.1)	3.0 (2.4–3.8)	2.2 (1.9–2.4)
	**Usual health care provider**								
	Yes	63,068	94.7 (94.1–95.3)	93.4 (92.4–94.3)	93.4 (92.4–94.3)	95.8 (95.4–96.2)	95.7 (94.7–96.5)	95.6 (95.0–96.2)	94.9 (94.5–95.3)
	No	3,491	5.3 (4.7–5.9)	6.6 (5.7–7.6)	6.6 (5.7–7.6)	4.2 (3.8–4.6)	4.3 (3.5–5.3)	4.4 (3.8–5.0)	5.1 (4.7–5.5)
	**Unmet health care need because of cost during past 12 mos.**								
	Yes	4,838	7.3 (6.7–8.0)	12.8 (11.4–14.3)	13.7 (12.5–14.9)	9.3 (8.7–10.0)	14.0 (12.3–15.9)	12.1 (10.9–13.4)	8.2 (7.7–8.7)
	No	61,761	92.7 (92.0–93.3)	87.2 (85.7–88.6)	86.4 (85.1–87.5)	90.7 (90.0–91.3)	86.0 (84.1–87.7)	87.9 (86.6–89.1)	91.8 (91.3–92.3)
	**Routine check-up within past 12 mos.**								
	Yes	58,551	90.1 (89.3–90.9)	89.0 (87.7–90.2)	89.0 (87.9–90.0)	91.0 (90.3–91.5)	90.1 (88.6–91.3)	89.4 (88.3–90.5)	90.2 (89.7–90.7)
	No	7,157	9.9 (9.1–10.7)	11.0 (9.8–12.3)	11.0 (10.0–12.1)	9.1 (8.5–9.7)	10.0 (8.7–11.4)	10.6 (9.5–11.7)	9.8 (9.3–10.3)

**Abbreviation:** CI = confidence interval.

* Respondents were asked “Are you deaf or do you have serious difficulty hearing?” (hearing); “Are you blind or do you have serious difficulty seeing, even when wearing glasses?” (vision); “Because of a physical, mental, or emotional condition, do you have serious difficulty concentrating, remembering, or making decisions?” (cognition); “Do you have serious difficulty walking or climbing stairs?” (mobility); “Do you have difficulty dressing or bathing?” (self-care); and “Because of a physical, mental, or emotional condition, do you have difficulty doing errands alone such as visiting a doctor’s office or shopping?” (independent living). Respondents who declined to answer, reported “don’t know,” and other missing responses were excluded from the analyses.

^†^ Unweighted sample size.

^§^ Each disability type might not be independent; a respondent might have two or more disability types.

## Discussion

This is the first report of disability prevalence measured using the U.S. Department of Health and Human Services six-question set through BRFSS and that examines sociodemographic characteristics and disparities in health care access by age group and disability type. In 2016, one in four noninstitutionalized U.S. adults reported any disability; a previous CDC report found a disability in one in five U.S. adults (*2*). The higher disability prevalence reported here likely resulted from the addition of the hearing disability question in 2016. The reported prevalence of hearing disability (5.9%) is consistent with other reports (*3*–*5*), and there were negligible (i.e., <1%) increases in prevalences of the other five disability types from 2013 to 2016.

Social determinants of health, such as sex, race/ethnicity, socioeconomic status, geographic location, and access to and use of quality health services influence the health and well-being of populations (*6*). Consistent with previous research (*2*), this analysis identified disparities in prevalences of any disability and disability type by sex, race/ethnicity, socioeconomic status, and geographic region. Women reported higher prevalences of any disability and of each disability type (except hearing and self-care) than did men. Higher prevalences of disability were reported by persons living in poverty; middle-aged adults living in poverty reported nearly five times the prevalence of mobility disability as did those who reported household income ≥200% of FPL. In this study, persons residing in the South U.S. Census region generally reported higher prevalences of disability. Chronic conditions associated with leading causes of disability (i.e., arthritis and heart trouble) (*7*) and associated lifestyle factors (e.g., smoking, overweight and obesity, and hypertension), are more prevalent in the South than in other U.S. Census regions.^¶¶^ The multiple determinants of health underscore the need for cross-sector approaches to effectively mitigate health inequities experienced by persons with disabilities.

Similar to previous research (*8*,*9*), this analysis identified disability-specific disparities in health care access, particularly among young and middle-aged adults. Disability-specific factors, such as severity of disability, age at disability onset, or having multiple disability types or comorbidities might partially explain why persons in these age groups, and those reporting self-care and mobility disabilities, had higher prevalences of access to care than did those reporting vision and hearing disabilities (*5*,*9*). Among persons aged ≥65 years, the primary disparity was in unmet health care need because of cost; adults reporting self-care disability had nearly twice the prevalence of cost-related unmet health care need than did those reporting hearing disability. By age 65 years, approximately 98% of Americans have access to Medicare coverage (*10*) and might have increased access to health care services. Nonetheless, older adults reporting self-care disability might face more financial strain because of a higher level of medical need compared with persons without such disability (*1*).

The findings in this report are subject to at least four limitations. First, BRFSS data are cross-sectional, and causality among sociodemographic characteristics, health care access, and disability cannot be inferred. Second, disability estimates are likely underestimates because BRFSS is only administered to noninstitutionalized adults and excludes persons living in long-term care facilities, such as older adults who might have higher disability prevalences. This could, in part, explain the higher prevalence estimates of cognitive disability among middle-aged and young adults compared with older adults, and the similar estimates of vision disability and self-care disability among middle-aged and older adults. In addition, questions used to assess hearing, vision, cognition, and mobility disabilities were designed to capture serious difficulty in these basic actions; thus, adults with milder difficulties might not be identified. Third, BRFSS data were self-reported and might be subject to self-report biases. Finally, nonresponse bias remains a possibility, although the weighting methodology used by BRFSS adjusts for nonresponse bias.

Prevalence of disability varied by age group and sociodemographic characteristics. Health care access varied by age group and disability type. Identifying disparities in access to health care highlights disability types and selected demographic groups*** that might benefit most from interventions that improve health care access, receipt of needed health services, and coordinated care. These have the potential to improve health behaviors, prevent secondary conditions, delay the progression of disability, or, through early detection of disease, permit early intervention that might improve health outcomes. Improved understanding of disability-specific differences in health care access and the provision of medical care might improve the specificity and effectiveness of interventions, accessibility, and outreach to reduce disability-specific disparities in health care access.

**Summary**

**What is already known about this topic?**

In 2013, based on questions to assess five disability types (i.e., vision, cognition, mobility, self-care, and independent living), one in five U.S. adults reported a disability.

**What is added by this report?**

In 2016, using the U.S. Department of Health and Human Services six-question set, one in four (61 million) U.S. adults reported any disability; nearly 6% reported hearing disability. Adults with disabilities, particularly those aged 18–44 and 45–64 years, experienced disparities in health care access by disability type.

**What are the implications for public health practice?**

Public health programs might benefit from the information provided in this report to develop and improve interventions, accessibility, and outreach to reduce disparities in health care access.

## Footnotes

* Based on Section 4302 of the Affordable Care Act, the U.S. Department of Health and Human Services issued data collection standard guidance to include a standard set of disability identifiers in all national population health surveys. https://aspe.hhs.gov/datacncl/standards/aca/4302/index.pdf.

† https://www.cdc.gov/brfss/.

§ Response rates for BRFSS are calculated using the standard set by the American Association for Public Opinion Research response rate formula 4 (http://www.aapor.org/AAPOR_Main/media/publications/Standard-Definitions20169theditionfinal.pdf). The response rate is the number of respondents who completed the survey as a proportion of all eligible and likely eligible persons. https://www.cdc.gov/brfss/annual_data/2016/pdf/2016-sdqr.pdf.

¶ The interviewer first reads a preamble to the telephone survey respondent (“The following questions are about health problems or impairments you may have. Some people who are deaf or have serious difficulty hearing may or may not use equipment to communicate by phone.”), followed by the six specific disability type questions. The questions are “Are you deaf or do you have serious difficulty hearing?” (hearing); “Are you blind or do you have serious difficulty seeing, even when wearing glasses?” (vision); “Because of a physical, mental, or emotional condition, do you have serious difficulty concentrating, remembering, or making decisions?” (cognition); “Do you have serious difficulty walking or climbing stairs?” (mobility); “Do you have difficulty dressing or bathing?” (self-care); and “Because of a physical, mental, or emotional condition, do you have difficulty doing errands alone such as visiting a doctor’s office or shopping?” (independent living).

** Health insurance coverage was ascertained by a “yes” response to the question “Do you have any kind of health care coverage, including health insurance, prepaid plans such as health maintenance organizations, government plans such as Medicare, or Indian Health Service?” Having a usual health care provider was assessed first with the question “Do you have one person you think of as your personal doctor or health care provider?” Persons who responded “no” were asked the question “Is there more than one, or is there no person who you think of as your personal doctor or health care provider?” Responses for having a usual health care provider were dichotomized into one or more and none. Receipt of a routine check-up was assessed with the question “About how long has it been since you last visited a doctor for a routine checkup? A routine checkup is a general physical exam, not an exam for a specific injury, illness, or condition.” Responses for having had a routine check-up within the preceding 12 months were dichotomized into within the past year or not within the past year. Unmet health care need because of cost was ascertained by a “yes” response to the question “Was there a time in the past 12 months when you needed to see a doctor but could not because of cost?”

†† Persons in all racial groups were non-Hispanic. Persons who self-identified as Hispanic might have been of any race.

§§ Poverty categories are based on the ratio of the respondent’s annual household income to the appropriate simplified 2015 federal poverty threshold (given family size: number of adults (1–14) in the household and number of children (≥0) in the household) defined by the U.S. Census Bureau. This ratio is multiplied by 100 to be expressed as a percentage, and federal poverty thresholds were then used to categorize respondents into four FPL categories: 1) <100% of FPL (poor), 2) ≥100%–<200% of FPL (near poor), 3) ≥200% of FPL (not poor), and 4) unknown.

*** Disability and Health Data System (https://dhds.cdc.gov/), an online, interactive data tool developed and maintained by CDC, presents yearly state-level data on prevalence of disability as well as approximately 30 demographic and health indicators, including health care access, for adults with disabilities overall and by type.
